# T1121G Point Mutation in the Mitochondrial Gene *COX1* Suppresses a Null Mutation in *ATP23* Required for the Assembly of Yeast Mitochondrial ATP Synthase

**DOI:** 10.3390/ijms23042327

**Published:** 2022-02-19

**Authors:** Guangying Yang, Tong Zhao, Shan Lu, Jun Weng, Xiaomei Zeng

**Affiliations:** Key Laboratory of Molecular Biophysics of Chinese Ministry of Education, Center for Human Genome Research, College of Life Science and Technology, Huazhong University of Science and Technology, Wuhan 430074, China; d201780464@hust.edu.cn (G.Y.); zhaot19931224@163.com (T.Z.); m201971907@hust.edu.cn (S.L.); jweng@hust.edu.cn (J.W.)

**Keywords:** *ATP23*, revertant, *COX1*, point mutation, mitochondrial ATP synthase, stability of Atp6

## Abstract

Nuclear-encoded Atp23 was previously shown to have dual functions, including processing the yeast Atp6 precursor and assisting the assembly of yeast mitochondrial ATP synthase. However, it remains unknown whether there are genes functionally complementary to *ATP23* to rescue *atp23* null mutant. In the present paper, we screen and characterize three revertants of *atp23* null mutant and reveal a T1121G point mutation in the mitochondrial gene *COX1* coding sequence, which leads to Val374Gly mutation in Cox1, the suppressor in the revertants. This was verified further by the partial restoration of mitochondrial ATP synthase assembly in *atp23* null mutant transformed with exogenous hybrid *COX1* *T1121G* mutant plasmid. The predicted tertiary structure of the Cox1 p.Val374Gly mutation showed no obvious difference from wild-type Cox1. By further chase labeling with isotope [^35^S]-methionine, we found that the stability of Atp6 of ATP synthase increased in the revertants compared with the *atp23* null mutant. Taking all the data together, we revealed that the T1121G point mutation of mitochondrial gene *COX1* could partially restore the unassembly of mitochondrial ATP synthase in *atp23* null mutant by increasing the stability of Atp6. Therefore, this study uncovers a gene that is partially functionally complementary to *ATP23* to rescue *ATP23* deficiency, broadening our understanding of the relationship between yeast the cytochrome c oxidase complex and mitochondrial ATP synthase complex.

## 1. Introduction

The mitochondrial respiratory chain (complexes I to IV) couples electron transfer to the generation of proton gradient, which is then dissipated via complex V (ATP synthase) to produce ATP [1]. Mitochondrial ATP synthase (ATPase) is a key enzyme catalyzing the synthesis of ATP in eukaryotic cells. Yeast mitochondrial ATPase is a multiprotein complex comprising a membrane-spanning complex (Fo) and a soluble component (F_1_), which generates or hydrolyzes ATP through the action of a rotational mechanism. The biogenesis and assembly of yeast mitochondrial ATP synthase are regulated by many nuclear gene products [2], among which some nuclear-encoded proteins, such as Nca2, Nca3, Atp22, Smt1, Atp10, and Atp23, have been implicated in the expression of subunit 6 (Atp6) and its interaction with the subunit 9 (Atp9) ring [3]. Nuclear-encoded Atp23 is a metalloprotease that has dual functions, including processing the yeast Atp6 precursor and assisting the assembly of ATP synthase [4,5]. In the absence of Atp23, newly synthesized Atp6 cannot be changed into mature Atp6, and mitochondrial ATP synthase cannot be assembled [4], indicating the essential role of Atp23 in the assembly of yeast mitochondrial ATP synthase. However, it remains unknown whether there are genes functionally complementary to *ATP23* to rescue *atp23* deletion mutant.

The cytochrome c oxidase (COX) complex is the terminal enzyme of the mitochondrial respiratory chains. This enzyme couples the transference of electrons from cytochrome c to oxygen with the translocation of protons from the matrix to the intermembrane space [6]. In *Saccharomyces cerevisiae*, the core of the enzyme is formed by subunits Cox1, Cox2, and Cox3, which are encoded by mitochondrial DNA, and the remaining subunits of the COX complex are encoded by nuclear DNA [7]. Cox1 (subunit 1 of COX complex) is the largest subunit of the COX complex, with twelve transmembrane α-helices making up the bulk of the protein [6]. When the COX complex assembly is blocked by mutations on either structural subunits or accessory chaperones, Cox1 synthesis is observed to be down-regulated [8,9]. Moreover, Cox1 can stabilize the biosynthesis of subunits Cox2 (subunit 2 of COX complex) and Cox3 (subunit 3 of COX complex), indicating that Cox1 plays an important role in COX complex assembly [10].

In *Saccharomyces cerevisiae*, mitochondrial genes *COX1*, *ATP8* (coding subunit 8 of ATP synthase) and *ATP6* are co-transcribed as polycistronic [11]. It has been shown that normally assembled mitochondrial ATP synthase is indispensable for COX complex biosynthesis and assembly [12]. Mutations in some structural genes of mitochondrial ATP synthase in *Saccharomyces cerevisiae*, including *ATP1* (coding α subunit of ATP synthase), *ATP2* (coding β subunit of ATP synthase), *ATP4* (coding subunit b of ATP synthase), *ATP6*, *ATP7* (coding subunit d of ATP synthase), *ATP8*, and *ATP9*, have been reported to affect COX biogenesis or assembly [13,14,15,16,17]. The loss of the mitochondrial ATP synthase complex and/or its enzyme activity can have a direct negative impact on the assembly of COX complex [18,19,20]. The existence of high-molecular-weight complexes composed of Atp9 and Cox6 (subunit 6 of COX complex) in yeast mitochondria has also been reported. Deletion of Cox6 leads to reduced Atp9 ring formation and ATP synthase assembly [21]. Further characterization revealed that the Atp9-Cox6 complex is the source of Atp9 ring formation, and it was proposed that the complex may play a role in coordinating assembly and maintaining proper stoichiometry of the two oxidative phosphorylation enzymes [22].

In the present study, by screening and characterizing revertants of *atp23* null mutant, we reveal that a T1121G point mutation in the mitochondrial gene *COX1* coding sequence (p.Val374Gly mutation in Cox1 protein) of cytochrome c oxidase could partially restore the unassembly of yeast mitochondrial ATP synthase due to nuclear gene *ATP23* deficiency. Furthermore, we found that the stability of Atp6 of mitochondrial ATP synthase increased in the revertants. These results indicate that the T1121G point mutation in the mitochondrial gene *COX1* could partially suppress *ATP23* null mutation by increasing the stability of Atp6 of mitochondrial ATPase. Taking all the data together, our study uncovers a gene that is partially functionally complementary to *ATP23*, broadening our understanding of the relationship between the yeast cytochrome c oxidase complex and mitochondrial ATP synthase complex.

## 2. Results

### 2.1. Isolation and Characterization of Revertants of Atp23 Null Mutant

**Figure 1:**
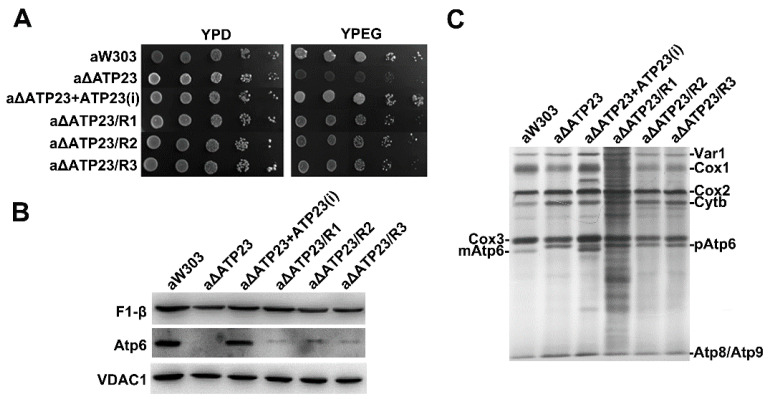
Growth properties and subunit 6 expression of *atp23* revertants. (**A**) Serial dilutions wild-type strain aW303, *atp23* null mutant (aΔATP23), *atp23* null mutant expressing chromosomally integrated copy of *ATP23* [aΔATP23 + ATP23(i)], and three revertants (aΔATP23/R1, aΔATP23/R2, aΔATP23/R3) were spotted on YPD (rich glucose) and YPEG (rich ethanol/glycerol) plates. Images were taken after growth for 2 days at 30 °C. (**B**) Western blot analysis of mitochondrial β subunit of F_1_ (F_1_-β) and Atp6 of Fo. Mitochondria (40 μg) prepared from same strains shown in (**A**) were analyzed by SDS-PAGE and immunoblotted with antibodies against F_1_-β, Atp6, and VDAC1. VDAC1 was used as a loading control. (**C**) In vivo labeling of newly synthesized mitochondrial translation products of revertants. Mitochondrial translation products in the same strains shown in (**A**) were labeled with [^35^S]-methionine in the presence of cycloheximide. The labeled mitochondrial translation products identified in the margin are ribosomal protein Var1, subunit 1 (Cox1), subunit 2 (Cox2), subunit 3 (Cox3) of cytochrome oxidase, cytochrome b (Cyt.b), and subunit 6 (mAtp6), subunit 8 (Atp8), and subunit 9 (Atp9) of ATPase. Precursor form of subunit 6 (pAtp6p) seen in the null mutant and revertants migrated slightly higher than the mature protein (mAtp6).

*Atp23* null mutant (aW303ΔATP23) cannot grow on non-fermentable carbon sources, such as ethanol and glycerol. Spontaneous revertants of aW303ΔATP23 were obtained by spreading the null mutant on non-fermentable carbon source medium (YPEG medium). The revertant colonies became discernible after 4–5 days. Three revertants (aΔATP23/R1, aΔATP23/R2, aΔATP23/R3) were isolated, and their growth on YPEG medium was compared to that of the *atp23* null mutant and the parental wild-type strain. Unlike the *atp23* null mutant, which had a very clear growth defect on YPEG, the three revertants grew at a rate considerably slower than that of the parental wild type (Figure 1A). 

To ascertain whether the three revertants could assemble a functional ATP synthase (F_1_-Fo) complex, several criteria were applied. The sensitivity of mitochondrial ATPase activity to oligomycin makes it a simple and reliable indicator of the presence of a functional F_1_-Fo complex. The results show that oligomycin treatment leads to 35–50% inhibition of mitochondrial ATPase activity in the revertants, but has no effect in *atp23* null mutant, indicating that the sensitivity of mitochondrial ATPase activity to oligomycin is partially restored in the revertants (Table 1). The presence of Fo of ATPase is also supported by the presence of a little Atp6 of mitochondrial ATP synthase in the revertants, although at a much lower level than that in wild type (Figure 1B). All of these results suggest that the assembly of functional mitochondrial ATP synthase is partially restored in the revertants.

Atp23 has the dual functions of processing yeast Atp6 precursor and assisting assembly of mitochondrial ATP synthase, but proteolytically inactive *atp23* mutant with Atp6p precursor was shown to be able to assemble the functional ATPase complex [4]. To determine whether the function of processing Atp6 precursor was also recovered in the revertants, newly synthesized mitochondrial translation products were investigated by isotope labeling. Similar to *atp23* null mutant, only Atp6 precursor (pAtp6), but not mature protein, was detected in the revertants (Figure 1C), indicating that only the function of assisting the assembly of ATPase of Atp23 was recovered in the revertants. 

### 2.2. The Suppressor of the Revertants Was Localized to T1121G Point Mutation of Mitochondrial Gene COX1 Coding Sequence

**Figure 2:**
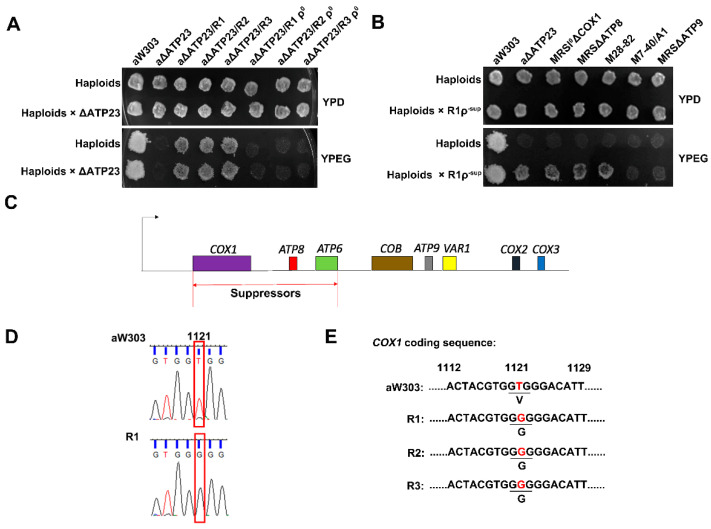
Locating the suppressor(s) in revertants. (**A**) Growth of diploids that revertants (R1, R2, R3) or ρ^0^ of the revertants (R1ρ^0^, R2ρ^0^, R3ρ^0^) crossed to *atp23* null mutant on YPD or YPEG medium. ρ^0^ colonies with complete deletion of mitochondrial DNA were obtained from revertants treated with ethidium bromide. (**B**) Growth of ρ^−sup^ colonies crossed to *atp23* null mutant and mitochondrial DNA mutant testers on YPD and YPEG medium. ρ^−sup^ represents ρ^−^ colony with partial mitochondrial DNA fragment containing the suppressor. M28-82 and M7-40/A1 are *atp6* and *cob* loss-of-function mutants, respectively (**C**) Diagram of localization of the suppressor(s) in mitochondrial DNA region of revertant R1. (**D**) Sequencing result of *COX1* coding sequence within mitochondrial DNA fragment in ρ^−sup^ colony derived from revertant R1. (**E**) Sequencing results of *COX1* coding sequence in mitochondrial genome of three revertants.

Next, we wondered where the suppressor(s) was (were) localized in the genome of the revertants. To investigate whether the suppressor(s) of the revertants were located in the mitochondrial DNA or nuclear DNA, the ρ^0^ mutants of the revertants, which lacked mitochondrial DNA, were generated and crossed to *atp23* null mutant. The diploid cells issued from the crosses fail to grow on the YPEG medium (Figure 2A), indicating that the suppressor(s) is (are) dominant mutations in mitochondrial DNA but not nuclear DNA. Then, the region of mitochondrial DNA responsible for suppressing the *atp23* null mutation was assessed by ρ^−^ deletion mapping. By treating the revertant R1 with ethidium bromide, we obtained a library of ρ^−^ colonies that lacked partial mitochondrial DNA. The purified ρ^−^ colonies were crossed to the *atp23* null mutant to select ρ^−^ with suppressor(s) colonies that could complement *atp23* null mutant. Then, we crossed ρ^−^ with suppressor(s) colonies to several mitochondrial DNA mutant testers, which contained loss-of-function point mutation or deletion mutation in mitochondrial genes, to localize the position of the suppressor(s) in the mitochondrial DNA of the revertants. The result showed that the ρ^−^ with suppressor(s) colonies could complement *cox1* mutant, *atp6* mutant and *atp8* mutant, but not *cob* or *atp9* mutant (Figure 2B), indicating that the ρ^−^ with the suppressor(s) colonies contains the mitochondrial DNA covering the region from *COX1* to *ATP8,* and the suppressor(s) in R1 should be localized to this region (Figure 2C). We further sequenced this region and found a single T1121G point mutation of *COX1* coding sequence (p.Val374Gly of Cox1) in the revertant R1 (Figure 2D), suggesting that this point mutation could be the suppressor in the revertant. 

To make sure the T1121G mutation in the *COX1* coding sequence is the only mutation in the mitochondrial DNA of the revertant as the responsible suppressor, we further sequenced the whole mitochondrial genome of the revertant R1. The sequencing results show that the T1121G mutation in the *COX1* coding sequence is the only mutation found in the revertant R1 mitochondrial genome (data not shown), while the rest of the mitochondrial DNA sequences are the same as the wild type. This was consistent with the above finding of *COX1* T1121G mutation in the ρ^−^ with suppressor(s) colonies of the revertant R1. To further confirm the suppressor site in all three revertants, the fragments containing the mutation site of *COX1* genome were amplified by PCR and sequenced in the three revertants (R1, R2, R3). The T1121G mutation of the *COX1* coding sequence is found in all three revertants (Figure 2E). These results indicate that the *COX1* T1121G point mutation (Cox1 p.Val374Gly mutation) should be the suppressor responsible for partial restoration of the mitochondrial ATP synthase assembly in the revertants.

### 2.3. The Suppressor Was Verified by Transformation of Exogenous Hybrid COX1 Plasmid in Atp23 Null Mutant

**Figure 3:**
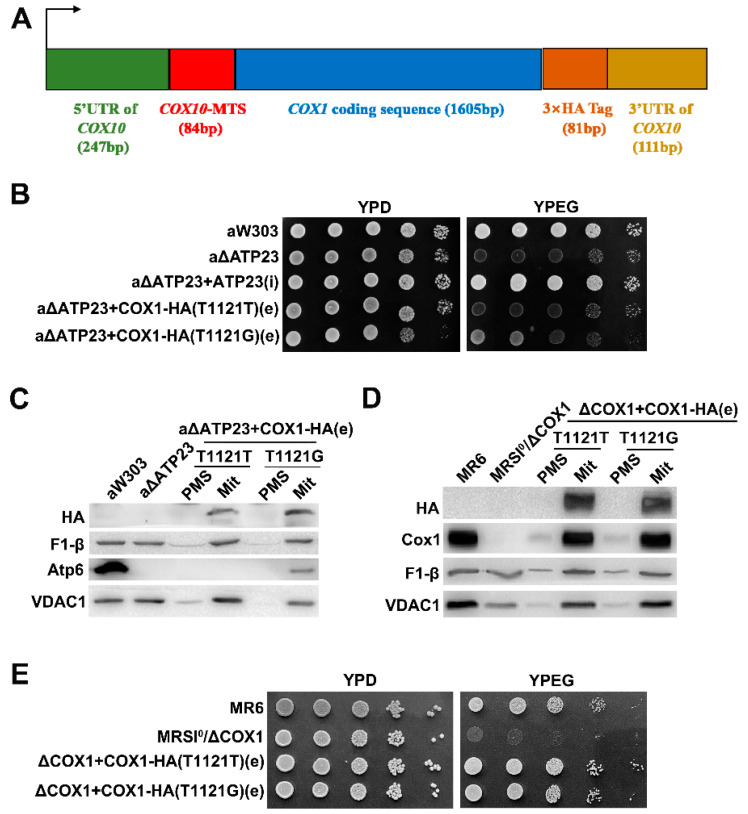
Verification of the suppressor by transformation of exogenous hybrid *COX1* construct in *atp23* null mutant. (**A**) Diagram of a hybrid *COX1-HA* plasmid construct. (**B**) Growth of *atp23* null mutant transformed with a hybrid *COX1-HA* construct plasmid on YPD and YPEG medium. aW303 and aATP23 + ATP23(i) were used as positive controls. (**C**) Western-blot analysis of localization of Cox1-HA protein (visualized by antibody against HA), mitochondrial β subunit of F_1_ and Atp6 of Fo. Mitochondria (Mit) and the postmitochondrial supernatant fraction (PMS) consisting mainly of cytosolic proteins were prepared from same strains shown in (**B**) except strain aATP23 + ATP23(i). Samples of mitochondria and PMS containing 40 μg protein were analyzed. (**D**) Western-blot analysis of the localization of Cox1-HA protein (visualized by antibody against HA and anti-Cox1), and mitochondrial β subunit of F_1_. Mitochondria (Mit) and post-mitochondrial supernatant fraction (PMS) were prepared from *cox1* null mutant transformed with hybrid *COX1-HA* construct plasmid. Samples of mitochondria and PMS containing 40 μg protein were analyzed. (**E**) Growth of *cox1* null mutant transformed with hybrid *COX1-HA* construct plasmid on YPD and YPEG medium. MR6 was used as a positive control.

The above results indicate that the T1121G mutation in the mitochondrial DNA *COX1* coding region should be the suppressor of the *atp23* null mutant. To further verify this, we constructed a hybrid *COX1-HA* plasmid, containing *COX1* coding sequence in the middle, with 5′ untranslated region (UTR) and mitochondrial targeting sequence (MTS) of nuclear gene *COX10* at 5′-terminus and three hemagglutinin tag (HA-tag) coding sequences and 3′-UTR of *COX10* at 3′-terminus (Figure 3A). It was supposed that the encoded hybrid protein was translated in cytoplasm and transported into mitochondria by the MTS of *COX10* for functions. *Atp23* null mutant was transformed with hybrid wild-type *COX1-HA (T1121T)* plasmid or hybrid mutant *COX1-HA (T1121G)* plasmid to investigate whether the *atp23* null mutant could be rescued. The results indicate that the transformant overexpressing hybrid wild-type *COX1-HA (T1121T)* plasmid fails to grow on the non-fermentable carbon source (YPEG) medium, as *atp23* null mutant did, while the transformant overexpressing hybrid *COX1-HA (T1121G)* mutant plasmid shows slow growth on the same medium (Figure 3B). Further subcellular localization investigations showed that both hybrid wild-type and mutant Cox1 were localized in mitochondria but not in the cytoplasm, the same as β subunit of F_1_. Moreover, stable Atp6 of mitochondrial ATP synthase was detected in the transformant overexpressing hybrid mutant *COX1-HA (T1121G)* plasmid, but not the hybrid wild-type *COX1-HA (T1121T)* plasmid (Figure 3C), suggesting that hybrid Cox1-HA proteins entered mitochondria successfully and the unassembly of mitochondrial ATP synthase of *atp23* null mutant could be partially rescued by hybrid mutant Cox1 protein. This was also confirmed by the partial recovery of sensitivity of mitochondrial ATPase activity to oligomycin in the *atp23* null mutant transformed with hybrid mutant *COX1-HA (T1121G)* plasmid (Table 2). The full functionality of the hybrid *COX1-HA* constructs is also confirmed by the localization of Cox1-HA protein in mitochondria (Figure 3D) and partial restoration of growth on the non-fermentable source of the *cox1* null mutant transformed with the constructs (Figure 3E). 

### 2.4. Cox1 p.Val374Gly Mutation Did Not Affect the Tertiary Structure of Cox1 Protein

**Figure 4:**
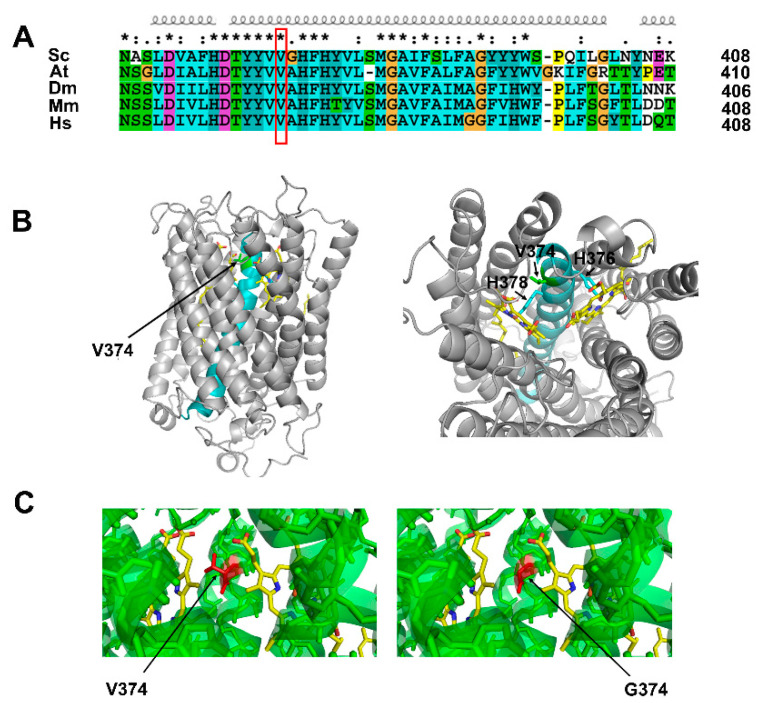
Cox1 p.Val374Gly mutation did not affect the protein tertiary structure. (**A**) Alignment of yeast Cox1 sequences containing Val374 in different species. Sequences of *Saccharomyces cerevisiae (Sc)*, *Aradopsis thaliana (At)*, *Drosophila melanogaster (Dm)*, *Mus musculus (Mm)*, and *Homo sapiens (Hs)* are aligned with ClustalX 2.1. Identical and conserved residues were marked with asterisks. Val374 is marked with a red rectangle, and the α-helices are marked on the top of sequences. (**B**) Side view (**left** panel) and top view (**right** panel) of Cox1 from *Saccharomyces cerevisiae* (PDB code: 6hu9). The transmembrane α-helix containing Val374 is colored in cyan, Val374 in green, His376 and His378 in blue, and two hemes in yellow. (**C**) Tertiary structure of wild-type yeast Cox1 (PDB code: 6hu9; **left** panel) and Cox1 p.Val374Gly mutation (predicted by SWISSMODEL; **right** panel). The transmembrane α-helix is colored in green, residue 374 in red, and two hemes in yellow.

The above results indicate that the T1121G mutation in the *COX1* gene, which encodes Val374Gly Cox1 mutant protein, could partially complement the *ATP23* deficiency. We wondered whether the Cox1 p.Val374Gly mutation affected its tertiary structure. By aligning Cox1 protein sequences from different species, it can be observed that the Val374 residue of Cox1 is located in the middle of the conserved transmembrane α-helix region, and is highly conserved from yeast to humans in eukaryotic organisms (Figure 4A). From the tertiary structure of yeast Cox1 (PDB code: 6hu9), we revealed that the transmembrane α-helix containing Val374 was buried in the center of Cox1, and Val374 is near two histidine sites, His376 and His378, which bind heme a3 and heme a, respectively (Figure 4B). Based on the yeast Cox1 structure (PDB code: 6hu9), the tertiary structures of Cox1 containing the p.Val374Gly mutation were predicted with SWISS-MODEL software, and the result shows that the mutation from valine to glycine at the 374 amino acid site has no significant impact on the surrounding spatial arrangement around residue 374 or the whole tertiary structure of Cox1 protein (Figure 4C). 

### 2.5. Stability of Atp6 Increased in the Revertants

**Figure 5:**
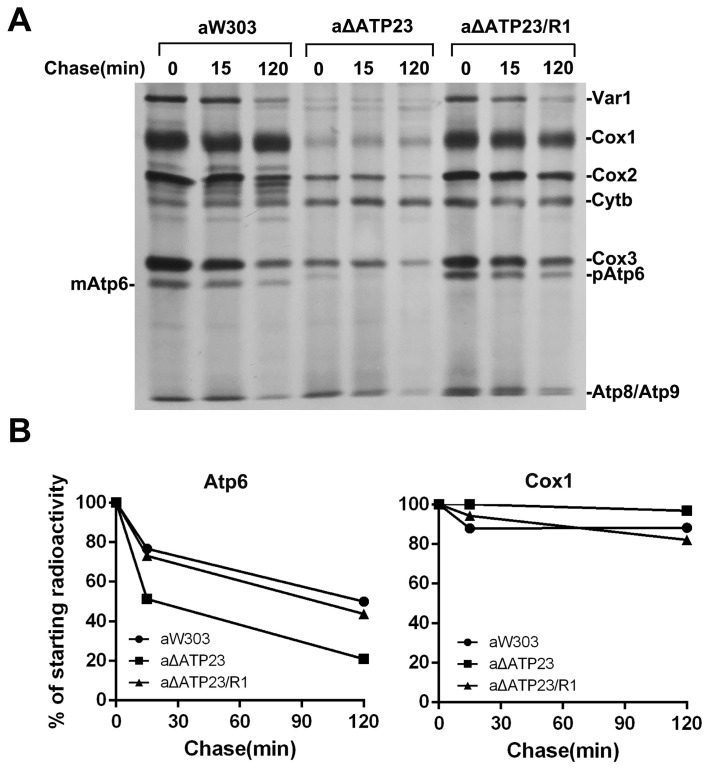
Increased stability of Atp6 in revertants. (**A**) Chasing stability of newly synthesized Atp6 by chasing labeling. Wild-type aW303, *atp23* null mutant aΔATP23, and *atp23* revertant aΔATP23/R1, were grown in YPGal and labeled in vivo for 20 min with [^35^S]-methionine in the presence of cycloheximide. Excess cold methionine was added to stop labeling, and samples were taken after the indicated time at 30 °C. (**B**) Quantification of the radioactivity of Atp6 and Cox1. The bands corresponding to Atp6 or Cox1 were quantified with a Phosphor Imager and normalized to value obtained at time zero.

Next, we wondered why the assembly of mitochondrial ATP synthase could be partially restored in the revertants. The stability of newly synthesized mitochondrial translation products was investigated by chasing labeling with isotope [^35^S]-methionine. After 120 min of chasing, as much as 50% of newly synthesized Atp6 was detected in the wild type and only about 20% in the *atp23* null mutant, but about 45% was detected in the revertants, indicating that the stability of Atp6 increased in the revertant compared with *atp23* null mutant, though it was still lower than in the wild type (Figure 5A,B). There was no obvious difference in the stability of Cox1 between the revertant and the *atp23* null mutant. This suggests that the Cox1 Val374Gly mutation results in increased stability of newly synthesized Atp6 precursor in the revertant, which enabled the Atp6 precursor partially assemble into functional mitochondrial ATP synthase. 

## 3. Discussion

Mitochondrial ATP synthase is composed of two functionally distinct sectors: F_1_ and Fo. Its assembly is a complex biological process. Previous studies found that nuclear-encoded Atp23 had dual functions of processing Atp6 precursor and assisting the assembly of yeast mitochondrial ATP synthase [4]. The metalloprotease motif (HEXXH) of yeast Atp23 is responsible for its function of processing Atp6 precursor [4,5], and residues 112–115 (LRDK) of Atp23 are responsible for its function in assisting the assembly of ATP synthase [23]. Moreover, the protein sequences of Atp23 are highly conserved in different species. However, it remains unknown whether there are genes functionally complementary to *ATP23* in yeast. In this study, we screened and characterized the revertants of *atp23* null mutant, and revealed that the suppressor in the revertant is encoded by mitochondrial DNA and the suppressor is a point mutation of T1121G in the mitochondrial gene *COX1* coding sequence, which results in a single amino acid mutation of Val374Gly of Cox1. This was verified by the partial restoration of assembly of mitochondrial ATP synthase in the *atp23* null mutant transformed with exogenous hybrid *COX1 T1121G* mutant construct plasmid. This is the first report showing that a single amino acid mutation in the mitochondrial DNA encoded Cox1 of cytochrome c oxidase could partially rescue the assembly of mitochondrial ATP synthase due to the deficiency of its assembly factor. Moreover, our results reveal that the partial rescue is due to the increased stability of Atp6 precursor of mitochondrial ATP synthase in the revertants. As mentioned above, the detection and characterization of the yeast Atp9-Cox6 complex revealed its role in the coordination assembly of the COX complex and ATP synthase complex [22,23]. Our study provides more evidence about co-regulation of these two complexes.

**Figure 6:**
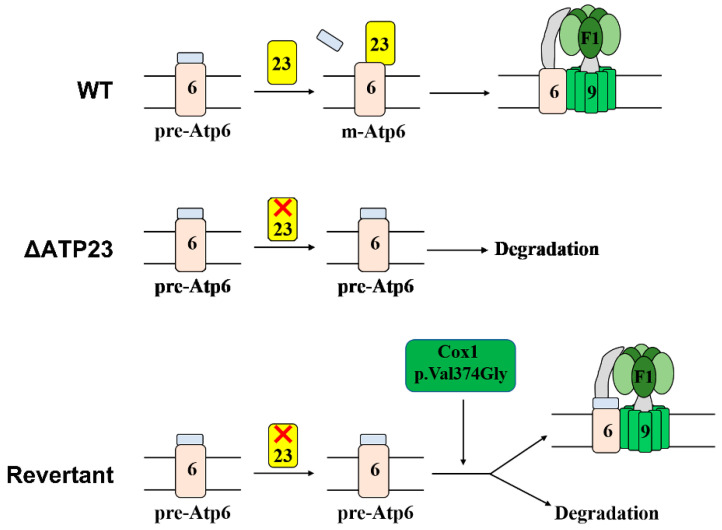
Model of the partial assembly of mitochondrial ATP synthase in revertant of *atp23* null mutant. The top panel shows that, in the presence of Atp23, Atp6 precursor (pre-Atp6) is processed into mature Atp6 (m-Atp6) and mitochondrial ATP synthase is assembled. The middle panel shows that, due to a lack of Atp23, the Atp6 precursor is degraded and no mitochondrial ATP synthase is assembled. The bottom panel shows that, in revertant of *atp23* null mutant, Cox1 p.Val374Gly point mutation stabilizes some pre-Atp6 and results in the partial assembly of the ATP synthase. Meanwhile, some pre-Atp6 is degraded.

According to previous studies, screening and analyzing revertants of the deletion mutant are good methods to study functionally complementary genes and the interactions between genes or proteins in yeast cells. Nuclear-encoded Atp10 regulates the assembly of yeast mitochondrial ATP synthase by physically interacting with newly translated Atp6 to promote assembly of Atp6 with the Atp9 ring [24]. By screening and characterizing the revertants of *atp10* null mutant, researchers revealed that the p.Ala249Val amino acid mutation of Atp6 was the suppressor of *ATP10* deletion, leading to the partial assembly of mitochondrial ATP synthase in the revertants [25]. *SHY1* is required for the expression of yeast Cox1. It was found that T167R and F199I mutations of Mss51, the specific translation factor of Cox1, can suppress *SHY1* null mutation by analyzing revertants of *SHY1* deletion mutant [26]. Interestingly, in our study, we revealed that a p.Val374Gly mutation of Cox1 could partially rescue *ATP23* deficiency by the screening and characterizing revertants of *atp23* null mutant. In the wild-type, Atp23 cleaves N-terminal leader peptide of the Atp6 precursor and assists the assembly of mature Atp6 into Atp9 ring to form the ATP synthase complex (top panel of Figure 6). In the absence of Atp23, the Atp6 precursor is unstable and is degraded, resulting in no assembly of ATP synthase (middle panel of Figure 6). In the revertants of *atp23* null mutant, due to Cox1 p.Val374Gly point mutation, the stability of Atp6 precursor increases, which promotes the partial assembly of mitochondrial ATP synthase (bottom panel of Figure 6). 

According to our investigation, in a co-immunoprecipitation experiment, Cox1 did not interact directly with Atp23 (data not shown). The p.Val374Gly mutation of Cox1 did not affect the tertiary structure of Cox1 protein but increased the stability of the Atp6 precursor in the revertants. The increased stability of the Atp6 precursor in the revertants was not due to the increased expression of Nam1, which has an important role in Atp6 stability, or of the molecular chaperone Atp10, which assists the assembly of Atp6 into mitochondrial ATP synthase (data not shown). During electron transfer, electrons are donated by cytochrome c to a dicopper centre (CuA) in Cox2 and transferred to the O_2_ reducing binuclear center (composed of heme a3 and a copper atom, CuB) in Cox1 via bis-histidine coordinated hemes [27]. According to the resolved yeast Cox1 structure, the Valine374 of Cox1 is localized in the middle of a transmembrane α-helix domain and close to the His376(H376) site (Figure 4C). The His376 in yeast Cox1 is a ligand of heme a3. It has been shown that H376A or H376N mutation leads to a complete lack of cytochrome c oxidase assembly, indicating that correct binding of the heme is required for assembly of the enzyme [28,29]. Thus, the Val374Gly mutation in the revertants of the *atp23* null mutant might affect the binding of histidine with heme and the assembly of the COX complex, leading to a lower electron transfer rate and resulting in the partial assembly of mitochondrial ATP synthase. The mechanism underlying the suppression of *atp23* null mutation by p.Val374Gly mutation of Cox1 is worth further study. 

Mutations in the core subunits of cytochrome c oxidase can lead to diseases. For example, the G6930A mutation in *COX1* coding sequence, resulting in a stop codon that causes the premature termination of translation with a predicted loss of 170 amino acids, can lead to multi-system mitochondrial disorders [30]. Thirty-eight types of mutations in the *COX1* gene were found in patients with familial adenomatous polyposis (FAP), and most of them were heteroplasmic changes of missense type (25/38) [31]. A missense T→A mutation at nucleotide position 7671 was identified in the *COX2* coding sequence, which affects the assembly or stability of the COX holoenzyme, leading to proximal myopathy and lactic acidosis [32]. Mutations in *COX3* were also reported to lead to complex IV deficiency and mitochondrial encephalopathy or mitochondrial myopathy [33,34]. To explore the possible methods of curing the diseases due to mitochondrial DNA, it was reported that transfecting mammalian cells with exogenously constructed target genes can rescue the loss of function due to mitochondrial gene mutations. For example, the transfection of exogenous hybrid mitochondrial gene *ND4* could rescue the protein assembly in complex I and prevent optic atrophy and visual loss due to *ND4* G11778A mutation [35]. In our study, for the first time, we reported that the product of exogenous hybrid mitochondrial *COX1* can be overexpressed in the cytoplasm and enter the mitochondria for certain functions, and the exogenous hybrid *COX1* T1121G mutation can partially restore the assembly of mitochondrial ATP synthase due to *ATP23* deficiency. This confirmed that Cox1 Val374Gly mutation can partially rescue *atp23* deficiency by restoring partial mitochondrial ATP synthase assembly. This also makes it possible to rescue yeast mitochondrial *COX1*, *COX2,* and *COX3* mutations by overexpressing hybrid constructs with a similar construction method in this study. Moreover, it also provides a new idea and method for treatment of human diseases, such as Alzheimer’s disease, myopathy, and cardiomyopathy, due to mitochondrial gene *COX1*, *COX2*, or *COX3* mutations. 

## 4. Materials and Methods

### 4.1. Yeast Strains and Growth Media 

The strains of *Saccharomyces cerevisiae* used in this study are listed in Table 3. The compositions of the medium for the growth of yeast were as follows: YPD (2% glucose 1% yeast extract, and 2% peptone); YPGal (2% galactose, 1% yeast extract, and 2% peptone); and YPEG (2% ethanol, 3% glycerol, 2% peptone, and 1% yeast extract). 

### 4.2. Serial Dilution Growth 

Cells were grown overnight in liquid YPD and the OD600 was adjusted to 1. Four times serial dilutions were made with distilled water, and 3 μL of each dilution was plated on YPD and YPEG plates and incubated at 30 °C for 2–3 days. 

### 4.3. Preparation of Yeast Mitochondria and ATPase Activity Assays

Mitochondria were prepared by the method of Faye et al. [39], except that zymolyase 20,000 instead of glusulase was used to convert cells to spheroplasts. The mitochondria concentration was determined by the Folin phenol procedure [40]. The ATPase activity of mitochondria was assayed by measuring the release of Pi from ATP in the presence or absence of oligomycin at 37 °C according to the procedure of King [41]. 

### 4.4. In Vivo and Chase Labeling of Mitochondrial Gene Products

For the in vivo labeling of newly synthesized mitochondrial translation products, 1 mL of cells grown overnight in YPGal media was collected, and the cells were resuspended and cultured in 10 mL of minimal galactose medium with the appropriate auxotrophic requirements for about 2 h. Cells equivalent to an OD600 of 0.5 were harvested at a growth density of OD600 of 1–2. After centrifugation and being washed with minimal galactose medium, the cells were suspended in 500 μL of the same buffer and 10 μL of a freshly prepared aqueous solution of cycloheximide (7.5 mg/mL) and incubated at 24 °C for 5 min. Then, 5 μL of [^35^S]-methionine (10 mCi/mL, PerkinElmer) was added and the cells were incubated at 24 °C for 30 min. After being centrifuged, the cells were resuspended with 500 μL of 20 mM cold methionine and 75 μL of 1.8 mM NaOH, 1 mM β-mercaptoethanol and 0.01 mM phenyl-methylsulfonyl fluoride (PMSF). Then, 575 μL of 50% TCA was added and the mixture was centrifuged. The precipitated proteins were washed once with 0.5 mM Tris (free base) and twice with water and were suspended in 45 μL of sample buffer [42]. Total cellular proteins were separated by SDS–PAGE on 12% polyacrylamide gel with 4 M urea and 25% glycerol. Proteins were transferred to a polyvinylidene fluoride membrane (Roche) and the radiolabeled mitochondrial gene products were visualized by autoradiography.

For the chase labeling of mitochondrial translation products, the cells were treated as above. After incubation with 5 μL of [^35^S]-methionine (10 mCi/mL, PerkinElmer) at 24 °C for 30 min, the reaction was stopped by the addition of 25 mM cold methionine. The cells were divided into three parts and incubated at 30 °C for 0, 15 and 120 min, respectively. Then, the samples were centrifuged and resuspended with 500 μL of 20 mM methionine and 75 μL of 1.8 mM NaOH, 1 mM β-mercaptoethanol and 0.01 mM PMSF and treated as above.

### 4.5. Mapping of Mitochondrial Suppressors

To investigate whether the suppressor(s) in the revertants was (were) mitochondrial DNA or nuclear DNA encoded, the revertant cells were treated with ethidium bromide and converted to ρ^0^ mutants [43]. Briefly, cells from *atp23* null mutant overnight cultures were spread at high density on respiratory substrates (YPEG medium). Revertant colonies became discernible after 4–5 days. Purified revertants were treated with ethidium bromide and converted to ρ^0^ mutants, then these purified ρ^0^ mutants were crossed to *atp23* null mutant, and the growth of diploids on respiratory medium was checked. 

To investigate the localization region of the suppressor in the revertants, the revertant cells were converted to ρ^−^ mutants with a lower concentration of ethidium bromide. Purified ρ^−^ mutants were crossed to the *atp23* null mutant and to mutants with mutations in the mitochondrial DNA *COX1*, *ATP6*, *ATP8, COB* and *ATP9* genes. The growth of diploids cells on respiratory medium was checked. 

### 4.6. Preparation of Mitochondrial DNA for Sequencing

Mitochondria were prepared by the method of Faye et al. [39]. Yeast mitochondrial DNA was further isolated from mitochondria by using a QIAprep Spin Miniprep Kit (Qiagen, Hilden, Germany) according to the manufacturer’s instructions. 

### 4.7. Construction of Hybrid COX1-HA Plasmid

The hybrid COX1-HA plasmid containing the COX1 coding sequence and its N-terminus was appended with 247 base pairs of 5′-UTR and the first 84 base pairs of COX10 corresponding to the mitochondrial targeting sequence (MTS). In addition, the coding sequences of 3 HA epitopes and 111 base pairs of 3’-UTR of COX10 were appended at the C-terminus of the COX1 coding sequence. As some mitochondrial codons are different from standard nuclear codons, 18 codons in the coding sequence of COX1 were changed to standard nuclear codons for expression in the cytoplasm. The codons of ATA in yeast mitochondrial COX1, which code Met in mitochondrial DNA but Ile in the cytoplasm, were changed to ATG. The codons of TGA in yeast mitochondrial COX1, which code Trp in mitochondrial DNA but are terminal codons in the cytoplasm, were changed to TGG. Codons CTA and CTT in yeast mitochondrial COX1, which code Thr in mitochondrial DNA but Leu in the cytoplasm, were changed to ACA and ACT respectively. The hybrid wild-type COX1-HA (T1121T) DNA was synthesized by Genewiz. The synthesized DNA was digested with BamH1 and HindIII and cloned into overexpression vector YEP352. The hybrid mutant plasmid COX1-HA (T1121G) was obtained by using the ClonExpress One Step Cloning Kit (Vazyme Biotech, Nanjing, China) according to the supplier’s instructions.

### 4.8. Miscellaneous Procedures

Standard methods were used for the preparation and ligation of DNA fragments and for transformation and recovery of plasmid DNA from *Escherichia coli* [44]. Yeast was transformed by the LiAc procedure of Schiestl and Gietz [45]. Proteins were separated by SDS–PAGE [42]. Western blots were treated with rabbit polyclonal antibodies against anti F_1_-β (a gift from Dr. Alexander Tzagoloff), anti-Atp6 (a gift from Dr. Jean-Paul di Rago and Dr. Marie-France Giraud), anti-HA (Biolegend, San Diego, CA, USA), and VDAC1 (Invitrogen, Waltham, MA, USA). The blots were then followed by a second reaction with peroxidase-coupled anti-rabbit IgG. The antibody complexes were visualized with the super signal chemiluminescent substrate kit (Pierce Chemical, Dallas, TX, USA). The protein concentrations were determined by the method of Lowry et al. [40].

## 5. Conclusions

This study uncovered that a point mutation in the mitochondrial gene *COX1* coding sequence could be partially functionally complementary to *ATP23* to rescue *ATP23* deficiency. We revealed that a T1121G point mutation in the *COX1* coding sequence was the suppressor in the revertants of *atp23* null mutant, which was further verified by the partially restored assembly of mitochondrial ATP synthase in the *atp23* null mutant transformed with exogenous hybrid *COX1 T1121G* mutant plasmid. Moreover, the suppression was due to increased stability of Atp6 in the revertants. This is the first report highlighting that a single amino acid mutation in mitochondrial DNA encoded Cox1 could partially rescue the assembly of mitochondrial ATP synthase due to a deficiency of its assembly factor. This broadens our understanding of the relationship between the yeast cytochrome c oxidase complex and mitochondrial ATP synthase complex. 

## Figures and Tables

**Table 1:** Mitochondrial ATPase activity and oligomycin sensitivity of revertants.

		ATPase Activity (μmol/mg/min)		
Strain	% ρ^+^	−Oligomycin	+Oligomycin	Inhibition (%)
aW303	>99	4.30	0.57	86.7
aΔATP23	22	1.54	1.26	18.2
aΔATP23 + ATP23(i)	>99	4.19	0.75	82.1
aΔATP23/R1	81	3.03	1.59	47.5
aΔATP23/R2	74	2.80	1.74	37.9
aΔATP23/R3	71	1.79	0.92	46.9

Mitochondria were prepared from wild-type, *atp23* null mutant, *atp23* null mutant expressing chromosomally integrated copy of *ATP23* [aΔATP23 + ATP23(i)], and *atp23* revertants grown on YPGal (rich galactose) medium. ATPase activity was measured at 37 °C.

**Table 2:** Mitochondrial ATPase activity and oligomycin sensitivity of transformants.

		ATPase Activity (μmol/mg/min)		
Strain	% ρ^+^	−Oligomycin	+Oligomycin	Inhibition (%)
aW303	>99	4.16	0.78	81.3
aΔATP23	25	1.58	1.35	16.5
aΔATP23 + ATP23(i)	>99	4.01	0.74	81.5
aΔATP23 + COX1-HA (T1121T) (e)	27	1.77	1.43	19.2
aΔATP23 + COX1-HA (T1121G) (e)	87	2.82	1.57	44.3

Mitochondria were prepared from wild type, *atp23* null mutant, *atp23* null mutant transformed with hybrid wild-type *COX1-HA (T1121T)* plasmid or mutant *COX1-HA (T1121G)* plasmid grown in YPGal medium. ATPase activity was measured at 37 °C.

**Table 3:** Genotypes and sources of yeast strains.

Strain	Genotype	Source
aW303	MATa *ade2-1 his3-1,15 leu2-3,112 trp1-1 ura3-1*	Dr. Rodney Rothstein ^a^
aW303ΔATP23	MATa *ade2-1 his3-1,15 leu2-3,112 trp1-1 ura3-1 atp23::HIS3*	[4]
W303ΔATP23	MATα *ade2-1 his3-1,15 leu2-3,112 trp1-1 ura3-1 atp23::HIS3*	[4]
aW303ΔATP23 + ATP23(i)	MATa *ade2-1 his3-1,15 leu2-3,112 trp1-1 ura3-1 atp23::HIS3 ATP23-HA*	[4]
aW303ΔATP23/R1, -R2, -R3	MATa *ade2-1 his3-1,15 leu2-3,112 trp1-1 ura3-1 atp23::HIS3 (ρ^+^^supR^)*	This study
aW303ΔATP23R1/ρ^−supR^	MATa *ade2-1 his3-1,15 leu2-3,112 trp1-1 ura3-1 atp23::HIS3 (ρ^−supR^)*	This study
M7-40/A1	MATα *ade1 cob*	[36]
M28-82	MATα *met6 Atp6*	[37]
MR6	ΜAΤa *ade2-1 his3-1,15 leu2-3,112 trp1-1 ura3-1 arg8::HIS3*	[38]
MRSI^0^ΔCOX1	MATα *ade1 his-1,15 leu2-3,112 trp1-1 ura3-1 arg8::HIS3 intronless cox1::ARG8^m^*	[38]
MRSΔATP8	MATα *ade2-1 leu2-3,112 trp1-1 ura3-1 arg8::URA3 atp8::ARG8^m^*	This study
MRSΔATP9	MATα *leu2Δ ura3-52 ade2-101 arg8::HIS3 atp9::ARG8^m^*	This study

^a^ Department of Human Genetics, Columbia University, New York.

## Data Availability

The data presented in this study are available in the article.
